# Supplementary material for: Building a Parkinson-Network–Experiences from Germany
Source: J Clin Med. 2020 Aug 25;9(9):2743. doi: 10.3390/jcm9092743 (PMC7563415; doi:10.3390/jcm9092743)
Supplement: Supplementary file 1 [file jcm-09-02743-s001.pdf]

Supplementary Material

Table S1. Overview of the Online Survey.

Page 1 [originally given in German],

Rating Parkinson Network Modules

Dear expert,
We are pleased that you have found the way to this survey. Parkinson networks are now widely represented in Germany. With a publication we would like to provide an overview of the various network modules (building blocks) in Germany and thus provide added value for existing and future networks. Your opinion is required!

The expert rating aims to round off the publication, so that the modules are not just presented, but also evaluated in terms of their effort and benefits. One module is briefly and clearly described per page. Below the description you will find five assessment categories, by means of which the modules can be assessed (each on a scale from 1 to 5). Please, evaluate the modules at your own discretion and experience.

The survey is written in English to enable consistency between the evaluation and the presentation of results, since the review work is to be published in an international journal. Participation is anonymous and non-binding.

If you have any questions, please contact munster@med.uni-marburg.de at any time.

We thank you for your participation and would be happy if you tell colleagues about the survey.

Best regards,
Prof. Dr. Carsten Eggers and Marlena van Munster
- Clinic for Neurology, University Hospital Gießen Marburg (Location Marburg) -

Do you agree to participate? (Then click on "Next") *
- ◯ yes
- ◯ no

Page 2

Page 2
Regional Demand Analysis/ Target Formulation

= joint, multidisciplinary, region-specific determination of needs and objectives

Aim:
• detect current issues, such as a lack of interface co-operation -> need-based goals can be derived (e.g. developing a communication platform)

Resources:
• No legal and financial resources are required (exception may be for renting a room and/ or catering)
• Time: required for target setting and communication with partners

Effort:
• Continuous integration of needs, target formulation and achievement (can take place in various forms, i.e. workshop)
• Optional: demand analysis with all partners at first meeting
• Optional: External representation (optional)

	none	little	some	much	very much	not applicable
additional workload	◯	◯	◯	◯	◯	◯
special expertise required	◯	◯	◯	◯	◯	◯
resources required for implementation expected	◯	◯	◯	◯	◯	◯
patient benefit	◯	◯	◯	◯	◯	◯
expected specialist benefit	◯	◯	◯	◯	◯	◯

Page 3

Page 3

Regional Public Relations

Aim:
• Create/ increase awareness of Parkinson´s disease (de-stigmatize); become notified by politics, possible sponsors, potential patients/ participants

Resources:
• Legal Framework: /
• Financing: /
• Time: required for meetings and organization

Effort:
• Initial: collecting relevant contact among network partners
• On-going: promoting the network and organizing publicity events (larger activities may require frequent meetings)

	none	little	some	much	very much	not applicable
additional workload	○	○	○	○	○	○
special expertise required	○	○	○	○	○	○
resources required for implementation expected	○	○	○	○	○	○
patient benefit	○	○	○	○	○	○
expected specialist benefit	○	○	○	○	○	○

Page 4

Page 4

Regional Utilities Atlas

= overview of regional care providers with expertise in Parkinson's disease

Aim:
• easy accessible and understandable information

Resources:
• Legal Framework: consent to store and map contact details
• Financing: required (depend on the presentation of the atlas)
• Time: required for collecting, mapping and updating data

Effort:
• Initial: reaching agreement among partners what/ who should be mapped, collecting relevant information, contacting care providers (consent)
• On-going: regular confirmation or adjustment of provider information (annually)
• External representation on homepage (consultation with legal- and data protection department required)

	none	little	some	much	very much	not applicable
additional workload	○	○	○	○	○	○
special expertise required	○	○	○	○	○	○
resources required for implementation expected	○	○	○	○	○	○
patient benefit	○	○	○	○	○	○
expected specialist benefit	○	○	○	○	○	○

Page 5

Page 5

Multidisciplinary Plenary Meetings

Aim:
- **Building trust**
- **regular exchange, joint decision making, documenting progress, stakeholder involvement and bundling ideas in a systematic way**

Resources:
- **Legal Framework: /**
- **Financing: may be required (speakers, room rental, catering and project management etc.)**
- **Time: depends on network, required for agenda creation and co-ordination (i.e. moderator for meetings)**

Effort:
- **steering team: agenda-setting, collecting proposals, inviting speakers and schedule new meetings**
- **partners: participation in meetings (approx. every 3 months)**
- **Optional: external communication (kick-off press release, project report etc.)**

	none	little	some	much	very much	not applicable
additional workload	○	○	○	○	○	○
special expertise required	○	○	○	○	○	○
resources required for implementation expected	○	○	○	○	○	○
patient benefit	○	○	○	○	○	○
expected specialist benefit	○	○	○	○	○	○

Page 6

Working Group

Aim:
- **processing needs & orders derived from network meetings, practice- and result-orientated solutions**

Resources:
- **Legal Framework: /**
- **Financing: Eventually (e.g. expert opinion, special workload for WG members).**
- **Time: depends on tasks and members**

Effort:
- **On-going collaboration between members when working on a task, (otherwise at rest)**
- **Optional: external presentation (i.e. on website, in presentations etc.)**

	none	little	some	much	very much	not applicable
additional workload	○	○	○	○	○	○
special expertise required	○	○	○	○	○	○
resources required for implementation expected	○	○	○	○	○	○
patient benefit	○	○	○	○	○	○
expected specialist benefit	○	○	○	○	○	○

Page 7

Steering Committee/ Project Management

= network management, control and organization

Aim:
• providing transparent & comprehensive information to the partners (maintaining motivation to participate), network organization

Resources:
• Legal Framework: /
• Financing: optional (e.g. for renting external project offices, meetings and catering)
• Time: required

Effort:
• Taking responsibility
• Frequent meetings (e.g. bi-monthly for the committee and weekly for the project management team)
• carefully communication with network partners
• External communication (i.e. online, press release etc.)

	none	little	some	much	very much	not applicable
additional workload	◯	◯	◯	◯	◯	◯
special expertise required	◯	◯	◯	◯	◯	◯
resources required for implementation expected	◯	◯	◯	◯	◯	◯
patient benefit	◯	◯	◯	◯	◯	◯
expected specialist benefit	◯	◯	◯	◯	◯	◯

Page 8

Homepage

Aim:
• Presenting the network to potential partners, patients and politics; informing & supporting other networks
• strengthen the commitment of partners

Resources:
• Legal Framework: Contract if a web designer is hired
• Financing: required if a web designer is hired
• Time: required for content creation and communication with the web designer

Effort:
• Initial: discussing, coordinating partners (obtaining logos, etc.), implementing the homepage
• Continuously: updating content, administrating the homepage
• Optional: drawing attention to the homepage (flyers, press releases, etc.)

	none	little	some	much	very much	not applicable
additional workload	◯	◯	◯	◯	◯	◯
special expertise required	◯	◯	◯	◯	◯	◯
resources required for implementation expected	◯	◯	◯	◯	◯	◯
patient benefit	◯	◯	◯	◯	◯	◯
expected specialist benefit	◯	◯	◯	◯	◯	◯

Page 9
Online Communication Plattform

Aim:
- easy communication between partners

Resources:
- Legal Framework: /
- Financing: required (approx. 80€/month for renting the platform)
- Time: required

Effort:
- enabling access for all partners
- setting up a basic structure
- facilitating regular use
- uploading/ updating content

	none	little	some	much	very much	not applicable
additional workload	◯	◯	◯	◯	◯	◯
special expertise required	◯	◯	◯	◯	◯	◯
resources required for implementation expected	◯	◯	◯	◯	◯	◯
patient benefit	◯	◯	◯	◯	◯	◯
expected specialist benefit	◯	◯	◯	◯	◯	◯

Page 10
Quickcards

= communication medium for care providers

Aim:
- promote interface communication within the network -> targeted and evidence-based therapy

Resources:
- Legal framework: contracts with printing company and graphic agency (optional) required
- Financing: printing costs of 100€ per card (approx.)
- Time: required

Effort:
- Initial: deciding on design and content of the cards together with network partners, communication with contract partners
- Later: annual meetings to discuss cards
- External presentation (i.e. at congresses), Inviting interested stakeholders to facilitate knowledge exchange and network growth.

	none	little	some	much	very much	not applicable
additional workload	◯	◯	◯	◯	◯	◯
special expertise required	◯	◯	◯	◯	◯	◯
resources required for implementation expected	◯	◯	◯	◯	◯	◯
patient benefit	◯	◯	◯	◯	◯	◯
expected specialist benefit	◯	◯	◯	◯	◯	◯

Page 11
Financing Strategies

Aim:
- secure long-term persistence of the network, fund projects, speakers, trainings or external partners (e.g. evaluation partners)

Resources:
- Legal Framework: eventually (i.e. when membership required)
- Financing: required (traveling, legal advice etc.)
- Time: required

Effort:
- Initial: analyze different strategies, present them to the network partners and discuss which paths should be followed and in which order
- Ongoing: frequent meetings (depends on the financing strategy(s) chosen)
- External communication to create transparency

	none	little	some	much	very much	not applicable
additional workload	○	○	○	○	○	○
special expertise required	○	○	○	○	○	○
resources required for implementation expected	○	○	○	○	○	○
patient benefit	○	○	○	○	○	○
expected specialist benefit	○	○	○	○	○	○

Page 12

Page 12
Evaluation

Aim:
- Investigating effectiveness (network as a whole or for single projects), measured against certain defined and transparent criteria

Resources:
- external partner required (e.g. research institute, university)
- Legal Framework: contract with partner
- Financing: required (previous experiences: 20.000€ to 70.000€)
- Time: required for co-ordination and parameter setting

Effort:
- Initial: establishing the target/ project or process to be evaluated and evaluation criteria (cooperation with external partner)
- Ongoing: frequent meetings, giving updates to partners
- Optional: external representation (i.e. public relations team may publish the results)

	none	little	some	much	very much	not applicable
additional workload	○	○	○	○	○	○
special expertise required	○	○	○	○	○	○
resources required for implementation expected	○	○	○	○	○	○
patient benefit	○	○	○	○	○	○
expected specialist benefit	○	○	○	○	○	○

Page 13

Page 13

Outpatient Video-supported Therapy

= Patient records short video sequences which are then evaluated by a neurologist who can obtain support from a clinic

Aim:
• real-life observation of symptoms, telephone contact with patient

Resources:
• Legal Framework: cooperation agreement between patient, neurologist and video device provider
• Financing: reimbursed by health insurance
• Time: required

Effort:
• learning handling of the technology
• within the German reimbursement scheme this service is once per year possible
• communicating service to patients/ network partners

	none	little	some	much	very much	not applicable
additional workload	○	○	○	○	○	○
special expertise required	○	○	○	○	○	○
resources required for implementation expected	○	○	○	○	○	○
patient benefit	○	○	○	○	○	○
expected specialist benefit	○	○	○	○	○	○

Page 14

Page 14

Structured Patient School according to the self-management concept

= combination of knowledge transfer and psycho-educative measures

Aim:
• strengthen participation and autonomy of Parkinson's patients (self-induced behavior modification)

Resources:
• Legal framework: written co-operation agreement
• Financial: required for external partners, content creation, meetings and communication
• Time: required

Effort:
• Creating content and discussion appropriate education strategies
• Acquiring patients
• Quality assessment

	none	little	some	much	very much	not applicable
additional workload	○	○	○	○	○	○
special expertise required	○	○	○	○	○	○
resources required for implementation expected	○	○	○	○	○	○
patient benefit	○	○	○	○	○	○
expected specialist benefit	○	○	○	○	○	○

Page 15

Page 15

Standardized Treatment Path

Aim:
• efficient, timely and equal care supply for all Parkinson patients

Resources:
• Legal Framework: co-operation agreement between care providers, health insurances etc.
• Financing: required (costs depend on the treatment path)
• Time: required

Effort:
• Initial: develop a consensus-based treatment path including the regional outpatient sector and under consideration of (inter-)national recommendations
• Co-ordinating tasks among partners
• Regular monitoring (3 months)

	none	little	some	much	very much	not applicable
additional workload	○	○	○	○	○	○
special expertise required	○	○	○	○	○	○
resources required for implementation expected	○	○	○	○	○	○
patient benefit	○	○	○	○	○	○
expected specialist benefit	○	○	○	○	○	○

Page 16

Page 16

Deep Brain Stimulation Consultation

= joint consultations practice owner - neurologist – patient

Aim:
• advice partner on advanced Parkinson´s syndrome and attract patients for deep brain stimulation and pump therapy

Resources:
• Legal Framework: /
• Financing: / (voluntary co-operation)
• Time: required

Effort:
• Making appointments via telephone agreement between practice and neurologists' secretariat

	none	little	some	much	very much	not applicable
additional workload	○	○	○	○	○	○
special expertise required	○	○	○	○	○	○
resources required for implementation expected	○	○	○	○	○	○
patient benefit	○	○	○	○	○	○
expected specialist benefit	○	○	○	○	○	○

Page 17

Page 17
Multimodal Complex Treatment

= In-patient treatment for patient
(minimum 16 days of inpatient treatment, multimodal assessment at beginning & end and 7.5 hours of therapy per week)
Aim:
- **increasing patient outcomes, safety & satisfaction, enhance loyalty, represent the facility as a center of competence**

Resources:
- **Legal Framework: /**
- **Financing: reimbursement by health insurance**
- **Time: weekly interdisciplinary team meetings**

Effort:
- **Patient registration**
- **Scheduling therapy sessions & weekly meetings via the case management**

	none	little	some	much	very much	not applicable
additional workload	○	○	○	○	○	○
special expertise required	○	○	○	○	○	○
resources required for implementation expected	○	○	○	○	○	○
patient benefit	○	○	○	○	○	○
expected specialist benefit	○	○	○	○	○	○

Page 18

Page 18
Rehab

= visits from the university hospital neurologist at the rehabilitation center to train staff and give advice

Aim:
- **expand competence regarding deep brain stimulation in external rehabilitation clinics and enhance patient safety & satisfaction**

Resources:
- **Legal Framework: /**
- **Financing: / (voluntary exchange)**
- **Time: required for meetings/ phone calls**

Effort:
- **making personal appointments w. external partner (external partner invites patient)**

	none	little	some	much	very much	not applicable
additional workload	○	○	○	○	○	○
special expertise required	○	○	○	○	○	○
resources required for implementation expected	○	○	○	○	○	○
patient benefit	○	○	○	○	○	○
expected specialist benefit	○	○	○	○	○	○

Page 19

Page 19

Virtual Visits

= electronic consultation of complex Parkinson patients (expert at university hospital – resident neurologist – patient) Aim:

• possibility to assign referrers and simplified communication with the patient

Resources:
• Legal Framework: provider needs to fulfill requirements of German eHealth act
• Financing: eventually for video camera
• Time: /

Effort:
• registration with a certified video consultation provider

	none	little	some	much	very much	not applicable
additional workload	○	○	○	○	○	○
special expertise required	○	○	○	○	○	○
resources required for implementation expected	○	○	○	○	○	○
patient benefit	○	○	○	○	○	○
expected specialist benefit	○	○	○	○	○	○

Page 20

Page 20

Parkinson Board

= opportunity for doctors in a specialized center to discuss complex patient cases (referral by resident neurologist) Aim:

• professional exchange, strengthening team communication and interaction with referrers

Resources:
• Legal Framework: /
• Financing: /
• Time: required for participation

Effort:
• Organizing meetings (by appointment or during weekly meetings)
• If neurologist is not present during discussion: sending recommendations for patient treatment afterwards

	none	little	some	much	very much	not applicable
additional workload	○	○	○	○	○	○
special expertise required	○	○	○	○	○	○
resources required for implementation expected	○	○	○	○	○	○
patient benefit	○	○	○	○	○	○
expected specialist benefit	○	○	○	○	○	○

Page 21

Page 21
Parkinson´s Info-Café

= informal exchange among patients, relatives and Parkinson Nurses

Aim:
• Strategic focus: giving advice on nursing aspects of the disease, creating patient loyalty towards the focus center

Resources:
• Parkinson staff, location, coffee and cake
• Legal Framework: /
• Financing: patient donations
• Time: required

Effort:
• Agenda Setting/ Organization (i.e. creating a fixed program item such as reading)
• Inviting participants (flyers and local press releases)

	none	little	some	much	very much	not applicable
additional workload	○	○	○	○	○	○
special expertise required	○	○	○	○	○	○
resources required for implementation expected	○	○	○	○	○	○
patient benefit	○	○	○	○	○	○
expected specialist benefit	○	○	○	○	○	○

Page 22

Page 22
PASS (Parkinson Assistant) Training

= curricular training for PASS

Aim:
• bring qualified, Parkinson-experienced medical assistants forward

Resources:
• Legal Framework: Co-operation contracts with external partners (training provider, participants) and legal agreement for sponsorship
• Financing: sponsorship, participation fee
• Time: /

Effort:
• Sending staff to the trainings (basic course: Fri/Sat on 2 weekends and advanced course 1 Sat. approx. 6 months later)
• Certificate for employees can be used for external representation

	none	little	some	much	very much	not applicable
additional workload	○	○	○	○	○	○
special expertise required	○	○	○	○	○	○
resources required for implementation expected	○	○	○	○	○	○
patient benefit	○	○	○	○	○	○
expected specialist benefit	○	○	○	○	○	○

Page 23

Page 23
Parkinson Nurse

Aim:
- competent care and advice for Parkinson patients
- Strategic focus: qualifying the nurse, increasing employee commitment and enabling qualified care

Resources:
- Legal Framework: /
- Financing: participation fee for educational courses
- Time: /

Effort:
- Assigning nursing staff to appropriate ward area

	none	little	some	much	very much	not applicable
additional workload	◯	◯	◯	◯	◯	◯
special expertise required	◯	◯	◯	◯	◯	◯
resources required for implementation expected	◯	◯	◯	◯	◯	◯
patient benefit	◯	◯	◯	◯	◯	◯
expected specialist benefit	◯	◯	◯	◯	◯	◯

Page 24

Page 24
Parkinson Academy

= practical training of care specialists (doctors, nurses, therapists)

Aim:
- expand specialist's knowledge together with co-operation partners & increase doctors´ attractiveness as referrer

Resources:
- Legal Framework: /
- Financing: sponsorship
- Time: required

Effort:
- Inviting participants
- Organizing agenda (practical training, a short theoretical update on current news, keynote speech & bedside teaching)
- Attracting sponsorship

	none	little	some	much	very much	not applicable
additional workload	◯	◯	◯	◯	◯	◯
special expertise required	◯	◯	◯	◯	◯	◯
resources required for implementation expected	◯	◯	◯	◯	◯	◯
patient benefit	◯	◯	◯	◯	◯	◯
expected specialist benefit	◯	◯	◯	◯	◯	◯

Page 25

Page 25

Parkinson Summer Festival

Aim:
- informal exchange between patients, relatives & specialists; patient recruitment for studies; increased patient retention

Resources:
- Legal Framework: /
- Financing: sponsorship & donations
- Time required for patient invitation & event planning

Effort:
- Planning the event with partners
- advertisement

	none	little	some	much	very much	not applicable
additional workload	◯	◯	◯	◯	◯	◯
special expertise required	◯	◯	◯	◯	◯	◯
resources required for implementation expected	◯	◯	◯	◯	◯	◯
patient benefit	◯	◯	◯	◯	◯	◯
expected specialist benefit	◯	◯	◯	◯	◯	◯

Page 26

Page 26

Symposium

Aim:
- scientific training of doctors, representing the facility as competent center

Resources:
- Legal Framework: /
- Financing: sponsorship
- Time: required (organization, invitation etc.)

Effort:
- agenda drafting prior to the event
- advertisement via flyers and on the university hospital homepage
- managing registrations

	none	little	some	much	very much	not applicable
additional workload	◯	◯	◯	◯	◯	◯
special expertise required	◯	◯	◯	◯	◯	◯
resources required for implementation expected	◯	◯	◯	◯	◯	◯
patient benefit	◯	◯	◯	◯	◯	◯
expected specialist benefit	◯	◯	◯	◯	◯	◯

Page 27 [originally given in German]

Do you have any final remarks?

Table S2. Overview of All Collected Parkinson Networks using the Template.

**Network:**	**Hamburg (Satellite Network Hamburg)**
Initiator and coordinating body:	Dr. med Becker & Dr. med. Wellach (Practice for Neurology & Psychiatry Hamburg Walddörfer)
Founding Year:	2011
Aim:	Improvement of the link between in- and outpatient care in Parkinson's disease in all phases of illness, optimized interface management (interdisciplinary, interprofessional and cross-sectoral)
Strategic priorities:	• interlink between in- and outpatient care through cross-sectoral medical activity, but also increased use of Parkinson nurses (PNs) and Parkinson's assistants (PASSes),<br>• networking with specialists, but also specialist clinics, university centers and professional groups in the field of nursing and activating therapies<br>• Inclusion of technical options / devices (e.g. sensors / video monitoring)
Participants among network partners:	currently: Practice for Neurology & Psychiatry HH Walddörfer, Ev. Amalie Sieveking Hospital, Clinic for Neurology and Clinic for Neurosurgery (THS, Prof. Hamel) and Nuclear Medicine (Mrs. Prof. Klutmann) UKE, incl. Ambulance Center for Parkinson's and Movement Disorders and Parkinson's Day Clinic (Prof. Buhmann), UKHS Lübeck (rare Parkinson's syndromes and genetics (Prof. Brüggemann), UKSH Kiel (Ms. Prof. Chr. Klein, Dr. Becktepe and Prof. Deuschl), various Parkinson's specialist clinics (Bad Segeberg, Prof. Hauptmann), various therapy facilities and specialist facilities, dPV (e.g. relatives group), QUANUP e.V. and others
Additional cooperation partners (contract based):	currently: Ev. Amalie Sieveking-Krankenhaus (EASK), UKE (under negotiation)
Legal framework:	Contractual (employer (EASK)), cooperations, association membership (QUANUP, board associated)
Financing:	currently, no regular external funding so far, some Funding through lectures, training and further education, individual funding projects
Structure of cooperation among partners:	Cooperation in terms of content in direct patient care, professional exchange, joint publications, advanced training events, Parkinson's nurses and PASSes
Communication:	Locally networked IT / KIS infrastructure, in writing (health insurance-Safenet), e-mail, by phone, directly (meeting)
Frequency:	Needs-based
External representation	Media, lecturing, training and further education, homepage
Network:	Lutherstadt (Parkinson Network Central Germany)
Initiator and coordinating body:	Alexianer Clinic Bosse Wittenberg, Clinic for Neurology, Initiated: Dr. Philipp Feige
Founding Year:	2009
Aim:	Ensuring and linking the outpatient with the inpatient care of Parkinson patients
Strategic priorities:	Establishment of the Parkinson's specialist clinic as an…<br>• in-patient provider,<br>• partner and advisor for the private practices, self-help groups, affected persons and relatives
Participants among network partners:	currently: Alexianer Clinic Bosse (Clinic for Neurology) in Lutherstadt Wittenberg as a "center" with resident neurologists and PD self-help groups in the city, surrounding districts in Saxony-Anhalt, Saxony and Brandenburg as well as the major centers Dessau, Halle / Saale and Leipzig
Additional cooperation partners (contract based):	Paul Gerhard Diakonie KH Wittenberg, radiological practice at the Alexianer Klink, Otto von Guericke University Magdeburg (Clinic for Sterotaxy), German Parkinson Association (DPV e.V.)
Legal framework:	Within the framework of German doctor - patient regulations

Financing:	additional costs only arise at national specialist and patient events that are supported by the pharmaceutical industry and clinic
Structure of cooperation among partners:	Clinic sees itself as a practitioner, consultant and 24-hour contact person. Equal cooperation with the network partners
Communication:	/
Frequency:	Patient event every two years, annual lectures in the clinic, external lectures several times a year in other clinics or with self-help groups in the network
External representation	Network flyer, which is updated annually, seal of quality (certification certificate) of the German Parkinson Association (DPV e.V.). since 2009 (every three years)
**Network:**	Parkinson Network Jena
Initiator and coordinating body:	Center for movement disorders University Hospital Jena
Founding Year:	2019
Aim:	Connecting in- and outpatient care facilities
Strategic priorities:	Optimizing case selection<br>Training of non-specialized neurologists, general practitioners and therapists<br>Patient empowerment
Participants among network partners:	University Hospital Jena<br>Eisenach Clinic (planned)<br>Stadtroda Specialist Clinic (planned)<br>Resident neurologists<br>Practices for physiotherapy, occupational therapy, speech therapy<br>Self-help groups
Additional cooperation partners (contract based):	/
Legal framework:	/
Financing:	Budget funds from University hospital
Structure of cooperation among partners:	Patient events / training<br>Continuing education<br>Training physio- / ergo- / speech therapy<br>Combined visits resident doctor/ clinician<br>Team meeting (clinic)<br>Team meeting (doctor-nurse)<br>Nurse consultation hours<br>Telemedicine<br>day clinic
Communication:	E-mail, phone, web portal (planned)
Frequency:	/
External representation	Homepage (under construction)
**Network:**	Düsseldorf
Initiator and coordinating body:	Düsseldorf University, Neurological Clinic, Department of Movement Disorders (Prof. Dr. Alfons Schnitzler); Coordinator Stefan Jun Groiss
Founding Year:	approx. 2010
Aim:	• Acquisition of patients for deep brain stimulation<br>joint consultation hours in practice as well as certified further training events for doctors in the Center for Movement Disorders and Neuromodulation of the University Clinic Düsseldorf the basis of the project
Strategic priorities:	• /

Participants among network partners:	• Senior physician at the university hospital, Institute for Clinical Neuroscience and Medical Psychology (Prof. Dr. Alfons Schnitzler), practice owner (i.e. Dr. med. Lorrain, Neuroärzte Düsseldorf)
Additional cooperation partners (contract based):	• none
Legal framework:	• informal appointments
Financing:	• Initially compensation for senior physicians through third party funding from the department. No more funds now. The practice owner did not receive any financial compensation.
Structure of cooperation among partners:	Collegial joint consultation<br>•
Communication:	/
Frequency:	Initially cooperation took place every 3 months (consultation hours in practices with a senior doctor of the clinic)<br>Then twice a year.
External representation	/
**Network:**	Münster and Osnabrück (Parkinson Network Münsterland+; PNM+)
Initiator and coordinating body:	University Hospital Münster (UKM)
Founding Year:	June 2017
Aim:	Optimizing care (chain) for people with Parkinson's disease and their families
Strategic priorities:	• Networking and exchange to make the care of people with Parkinson's more coordinated, patient-oriented and individual<br>• interdisciplinary care team with pooled expertise<br>Sharing experiences and building knowledge
Participants among network partners:	Clinics, general practitioners and specialists, physiotherapists, speech therapists, occupational therapists, pharmacists, rehabilitation, patients, relatives, self-help groups, Parkinson's nurses, Parkinson's assistants, nurses, industry<br>•
Additional cooperation partners (contract based):	See website: https://www.ukm.de/index.php?id=ukm-parkinsonnetz-muensterland
Legal framework:	Cooperation agreement as the basis of cooperation including working principles, rights and obligations (membership as an institution or individual possible)
Financing:	No membership fees<br>Initial phase financed by co-operation funds from AbbVie + Equity UKM<br><br>Future / long term planned:<br>• Funding<br>• Donate<br>• Sponsorship<br>UKM's own resources
Structure of cooperation among partners:	• Quarterly plenary meetings (all partners)<br>• Formation of working groups (focus groups): focus on internal coordination of meetings / coordination<br>Steering group
communication	• Online-Plattform
Frequency:	/

External representation	https://www.ukm.de/index.php?id=ukm-parkinsonnetz-muensterland
**Network:**	East Saxony (Parkinson Network East Saxony; PANOS)
Initiator and coordinating body:	PD. Dr. Martin Wolz, Meissen Elbland Clinic<br>Dr. Kai Loewenbrück, University Hospital Carl Gustav Carus Dresden<br>Dr. Peter Themann, Clinic at Tharandter Wald Hetzdorf
Founding Year:	2018
Aim:	• Establishment of a regional intersectoral structured treatment path for Parkinson patients with the need for specialized co-treatment<br>• Equal and timely involvement of patients with specialized treatment needs, regardless of where they live and other socio-demographic factors<br>Increase in quality of life related to illness, reduction of avoidable complications and unplanned emergency rooms
Strategic priorities:	Content implementation strategy<br>• Efficient and qualitatively uniform therapy based on a standardized intersectoral treatment path with the involvement of the outpatient medical sector<br>• Low barriers to entry thanks to low-threshold patient enrollment options for resident neurologists, general practitioners (in the area of East Saxony necessary with up to 40% exclusively primary care) and patients themselves<br>• Establishment of three regional specialized outpatient Parkinson centers (Dresden, Hetzdorf and Meißen) with a standardized path-based treatment concept<br>• Definition of an individual path-based further treatment concept by specialists at the Parkinson centers, graduation between 100% outpatient area and 100% specialized care at the centers<br>• Repetitive patient-based monitoring (every three months, initially via automated questionnaire management, and in the future also sensors), in order to be able to react in time to relevant changes regardless of the treatment concept.<br><br>Permanent technical, personnel and communication infrastructure:<br>• Establishment of an intersectoral path-supporting electronic documentation and patient management platform<br>• Establishment of a lifelong personal intersectoral case management ("Parkinson's Guide") as a contact and organizer of path-based therapy<br>• Active network management to mobilize private medical treatment partners and to implement continuous quality management<br>• Establishment of a standardized Parkinson patient school based on the Swedish National Parkinson School<br>• Establishment of a professional training and further education strategy<br>• Implementation of a structured growth concept (integration of further regions, centers and occupational groups, e.g. physiotherapy and occupational therapy, speech therapy)
Participants among network partners:	clinics:<br>• Meissen Elbland Clinic<br>• Clinic at the Tharandter Wald Hetzdorf<br>• University Hospital Carl Gustav Carus Dresden<br>Scientific institutes, companies:<br>• Institute for Applied Computer Science e.V., Leipzig (InfAI)<br>• Institute for Information Systems, TU Dresden<br>• What do I have? gGmbH<br>• Professorship for Movement Sciences, Chemnitz University of Technology<br>• Center for Medical Informatics, TU Dresden (ZMI)<br>Evaluation:<br>• Center for Evidence-Based Health Care (ZEGV), TU Dresden in cooperation with AOK PLUS Saxony-Thuringia<br>• Ikk Classic<br>Associations, professional organizations:<br>• German Parkinson's Association (dPV)<br>• Association of Statutory Health Insurance Physicians in Saxony (KVS)<br>Saxon State Medical Association (Slaek)
Additional cooperation partners (contract based):	see above, with the exception of dPV, Slaek, AOK PLUS and ikk Classic (all associated cooperation partners without their own budget)
Legal framework:	Health care research project

Financing:	German Fund for coal exit<br>= Combined funding from the Federal Ministry of Health and the Free State of Saxony
Structure of cooperation among partners:	Partnership based health services research project, written cooperation agreement
communication	• Active structured network management<br>• Workshop-based needs assessment<br>• Detailed planning of the treatment path based on an intersectoral multi-level consensus process with the involvement of health insurance companies and professional organizations<br>• Establishment of topic-related intersectoral working groups<br>Patient school, professional training and further education concept
Frequency:	• /
External representation	• Web presence under construction
**Network:**	Marburg (Parkinson network alliance Marburg; PANAMA)
Initiator and coordinating body:	Department of Neurology, Marburg University Hospital
Founding Year:	2016
Aim:	• Pooling activities related to Parkinson's disease<br>• Training and education of laypersons and specialists<br>Networking regional supply partners
Strategic priorities:	• Optimized collaboration with referrers<br>• Care of complex patients in the focus center<br>• identify suitable candidates for invasive therapies (DBS, pump therapies)<br>• enable the continued care of complex patients<br>• Scientific development of the Parkinson´s research area
Participants among network partners:	• BDH Clinic Braunfels<br>• Hardtwald Clinic Bad Zwesten<br>• Asklepios Clinic Fürstenhof Bad Wildungen<br>• Fulda Clinic<br>• Siegen district hospital<br>• Neurological therapy center and clinic for neurological and interdisciplinary early rehabilitation in the St. Marien Hospital Cologne<br>• Local community practice Reifschneider/ Unsorg/ Ries/ Schumann/ Knoblich/ Peters<br>• Tschirkova/ Matasaru<br>• Locations: Erbach, Groß-Umstadt, Darmstadt
Additional cooperation partners (contract based):	none
Legal framework:	none
Financing:	No regular funding so far, some funds through lectures (sponsorship) and trainings, single funding projects<br>• Donations mediated by the Neurology for Education, Research and Therapy Association
Structure of cooperation among partners:	• Joint training events in regional centers<br>• Joint visits to the local centers for the identification of patients for DBS/ pump therapy<br>Unstructured regular exchange
Communication:	No systematic communication channels<br>• Email/ Phone
Frequency:	/
External representation	Media, lecturing, training and further education, homepage: https://www.ukgm.de/ugm_2/deu/umr_neu/16242.html

Table S3. Overview of All Collected Network Modules using the Template.

Module name:	Regional Demand Analysis/ Target Formulation
Aim:	Overview of regional needs (e.g. lacking Interface communication/co-operation) deriving targets for the network (e.g. developing a communication platform)
Strategic priorities:	• Joint multidisciplinary region-specific determination of needs and objectives<br>• Prioritization of goals according to the likelihood of realization and the time required for realization (e.g. formulating the three most important ones)
Participants among network partners:	All
Additional cooperation partners (contract based):	none
Legal framework:	none
Financing:	No financing required<br>exception maybe for possible room rental and / or catering
Structure of cooperation among partners:	Moderated workshop; written query; Kick off<br>• Demand analysis: All partners<br>• Formulation of goals: core team, acceptance by partners at regular meetings<br>• Prioritization: core team
Frequency:	• Perform demand-analysis during the first meeting / foundation meeting if possible<br>• Continuously incooperate needs & requirements<br>• Continuously review target formulation & target achievement
External representation:	• Representation in the mission statement, if desired and available (e.g. framework etc.)<br>• Leaflet<br>• Preamble<br>• Press release
Module name:	**Multidisciplinary Plenary Meetings**
Aim:	• Getting to know each other in the region<br>• Regular exchange at eye level / building trust<br>• Voting on the results / suggestions for project groups<br>• Collecting new ideas / suggestions from partners
Strategic priorities:	• Knowledge transfer<br>• Documentation of progress<br>• Involvement of the project groups<br>• Systematic bundling and preparation of suggestions / ideas from the partners
Participants among network partners:	All partners, project team, moderation, record-taker
Additional cooperation partners (contract based):	none
Legal framework:	none
Financing:	Possibly<br>• Speakers<br>• room or catering costs<br>• external project management (if these activities cannot be carried out by partners)
Structure of cooperation among partners:	• Short telco within the core team<br>o Coordination of goals<br>o Determining the responsibility of the individual agenda items for the respective meeting<br>o Agenda creation and invitation<br>• Involvement of partners in proposals for the agenda<br>o Optional: Prepare presentation<br>o Optional: Prepare documents for distribution<br>• Scheduling the next meeting in the current meeting
Frequency:	• Every three months has proven itself

	• Project groups can meet in between
External representation:	• Kick-off press release<br>• Project report on completed topics and target achievement<br>• Participation in other indication-related or regional (health) events
Module name:	**Working Group**
Aim:	• Processing needs or orders derived from meetings with all partners<br>• Development of (solution) proposals for the plenary meetings with all partners
Strategic priorities:	• Practice- and result-oriented development of solutions<br>• "From the practice for the practice"
Participants among network partners:	Participants usually with special expertise / special interest in the topic
Additional cooperation partners (contract based):	none
Legal framework:	none
Financing:	• Generally, no costs other than room rental or catering<br>• Costs are only incurred in the case of a third-party assignment (e.g. expert opinion) or special workload on AG members (allowance for expenses)
Structure of cooperation among partners:	• WGs are put together at the multidisciplinary meeting at the request of the partners (expertise and voluntariness)<br>• Documentation of the results in short reports
Frequency:	• On an ongoing basis, if necessary, with information from the partners in the multidisciplinary plenary meeting<br>• Work only on tasks, otherwise the WG is at rest<br>• Project-related working groups are also possible (e.g. preparation of a press conference)
External representation:	• On the website<br>• In presentations<br>• Mentioned in the minutes of the multidisciplinary meetings
Module name:	**Homepage**
Aim:	• Attract attention from potential partners within the utilities<br>• Make the network available to patients<br>• Information about the network (participants, tasks, goals, achievements so far) can be obtained<br>• Provide support and exchange of experience for other networks / those interested in the network<br>• External presentation to politics and society<br>• Strengthen the commitment of the partners<br>• Show professionalism
Strategic priorities:	• Create an idea for a website<br>• Implementation with web designer (usually external)<br>• Standalone or subpage of an existing website
Participants among network partners:	Project team, working group and (external) web designer
Additional cooperation partners (contract based):	Contract with commissioned web designer
Legal framework:	contract
Financing:	depending on the design
Structure of cooperation among partners:	• Core network team discusses ideas with web designers<br>• Continuous implementation and support of the designers work by the network team<br>• Depending on the content, coordination with individual network partners may be necessary<br>• Can be time-consuming, as logos and consent e.g. for data must be obtained

Frequency:	• Continuously until completion (when going live)<br>• Afterwards: set into update mode, therefore publish as little dynamic data as possible<br>• Core-team tasks can be delegated to a working group
External representation:	• is part of the external presentation<br>• Draw attention in flyers, presentations, lectures, press releases
Module name:	**Regional Public Relations**
Aim:	• Achieve and increase awareness of Parkinson's<br>• De-stigmatization of the sick through education<br>• Make the network known in the region, including to generate new participants<br>• Be noticed by politics and possible sponsors<br>• Be noticed by potential patients<br>• To motivate the participants
Strategic priorities:	• Find the right contact person online<br>• Creation of information material
Participants among network partners:	• Partners, project team
Additional cooperation partners (contract based):	none
Legal framework:	none
Financing:	• Usually none, since the creation of information on the Internet and publication on the media of the network partners and their environment
Structure of cooperation among partners:	• During the first plenary meetings, ask for and list contact persons from the area of public relations and link addresses of the partners<br>• Find out urban and regional magazines and get in touch with those responsible<br>• Appoint "network spokesperson"<br>• Establish a working group "public relations"<br>• Organize publicity events
Frequency:	• ongoing.<br>• larger activities may need to be prepared during frequent meetings.
External representation:	• Regional public relations work is part of the external presentation
Module Name:	**Evaluation**
Aim:	Investigating the effectiveness of the entire network or single projects, measured against certain defined and transparent criteria
Strategic priorities:	• Definition of the target group of the evaluation<br>• Definition of the project to be evaluated<br>Definition of the appropriate parameters
Participants among network partners:	• Project team, working group "evaluation", external partner (e.g. research institute, university) required
Additional cooperation partners (contract based):	• partnership with an external partner
Legal framework:	• contract
Financing:	• from experience: between 20.000 and 70.000 €
Structure of cooperation among partners:	• Project team and working group define the target / project / process to be evaluated<br>• Development of evaluation criteria in cooperation with ext. partner<br>• External partners usually prepare the evaluation. based on questionnaires filled out by all partners<br>• Optional: Effective use of the evaluation results<br>• Updates at the plenary meetings
Frequency:	• frequent meetings during the evaluation<br>• Final presentation of the results in a plenary meeting
External representation:	• Use of evaluation results in the regional public, possibly also in specialist publications

	• Press release or press conference on the results of the evaluation
Module Name:	**Financing Strategy**
Aim:	• Long-term network security<br>• Financing of projects, speaker fees for training, information material, external partners (e.g. evaluation partners, web designers), catering, rentals etc.
Strategic priorities:	• Basic decisions (e.g. membership fees yes / no, cooperation with industry as a partner)<br>• Use of public funding<br>• Use of health insurance funding<br>• Determine the order for different financing ins
Participants among network partners:	• project team, all
Additional cooperation partners (contract based):	• Depending on the chosen form of financing<br>• (e.g. membership contracts, sponsorship contracts)
Legal framework:	• Usually contracts
Financing:	• Possibly traveling expenses
Structure of cooperation among partners:	• Project team analyzes different strategies and presents advantages and disadvantages<br>• Discussion with all partners and coordination, which paths should be followed and in which order<br>• In particular when using state funding, a separate WG should be founded
Frequency:	• Depending on the financing strategy (s) chosen
External representation:	• Create transparency vis-à-vis partners online and, if necessary, towards the public<br>• Government funding could also be used as advertising
Module Name:	**Steering Committee/ Project Management**
Aim:	Management, control and organization of the network
Strategic priorities:	• Takeover of project management internally by partner or commissioning an external project office<br>• Define responsibilities<br>• Planning meetings and content<br>• Detection of obstacles and "redirection" of the network<br>• Transparent and comprehensive information from the partners in order to maintain motivation and willingness to change<br>• Delegation of tasks<br>• Coordination of the working groups and their results
Participants among networkpartners:	• steering team, mutlidisciplinary
Additional cooperation partners (contract based):	none
Legal framework:	none
Financing:	• Possibly financing for external project offices depending on the volume of tasks, possibly limited in time or content<br>• Possibly room rental, catering for meetings<br>• Otherwise no financing is necessary
Structure of cooperation among partners:	• Regular meetings of the steering team<br>• Management and project management should e.g. vote weekly<br>• When communicating, consider all members of the team (e.g. "cc" in emails) to get the same level of information in the project team<br>• Important: willingness to take responsibility and make decisions
Frequency:	• Regular meetings (e.g. bi-monthly) for the steering group and e.g. weekly for project management (usually by phone)
External representation	• Presentation of the roles in the project team vis-à-vis the partners online, in press releases, on homepage and in presentations

Module Name:	**Online Communication Platform**
Aim:	Easy communication between all partners
Strategic priorities:	• joint planning of meetings<br>• Exchange of documents<br>• Collection of documents<br>• Possibility to contact individual partners for specific supply questions<br>• Conduct project discussions
Participants among network partners:	• steering committee, all
Additional cooperation partners (contract based):	none
Legal framework:	none
Financing:	• If existing tools are used, it is usually paying a rent for the platform, estimated at 80 euros / month
Structure of cooperation among partners:	• All partners have access to the platform<br>• All partners can participate in discussions and add contributions<br>• Possibly. Protected folders for the working groups and the steering committee (access only for the respective members)
Frequency:	• Permanent
External representation	• can be demonstrated in presentations (working method)<br>• otherwise not
Module Name:	**Regional Utilities Atlas**
Aim:	Overview of regional care providers with specific Parkinson's expertise
Strategic priorities:	• Compilation of all providers in the region including the specific Parkinson expertise<br>• Show utilities on a network card<br>• Describe targeted / specific assignments<br>• having the right contact person on the map<br>Gaps in supply, i.e. defile in the number of suppliers or contents in the region, become clear -> Countermeasures can be initiated
Participants among network partners:	• Project team, all
Additional cooperation partners (contract based):	none
Legal framework:	Not necessary,<br>• eventually approval that data may be stored (data protection)
Financing:	Depending on the presentation
Structure of cooperation among partners:	• Joint definition of the Parkinson-specific aspects of care which shall be mapped<br>• Written or digital query from all partners<br>• Regular confirmation or adjustment of the information by all partners
Frequency:	i.e. annually
External representation	• can be displayed on the homepage (consultation with legal department and data protection necessary!!)<br>• Or only for internal use by all partners
Module Name:	**Quickcards**
Aim:	Promotion of interface communication<br>Targeted referrals with additional information and standardized exchange -> targeted, evidence-based therapy
Strategic priorities:	• e.g. at the doctor / therapist interface: overview of the possible symptoms and associated therapy options (evidence-based), prepared in handy cards (quick cards)

	• Associated block, on which the respective finding / reason for referral can be checked (sheet is given to the patient) and the next provider can specifically align the therapy offer with this<br>• Can be implemented for all interfaces and relevant topics
Participants among network partners:	• Project team, working groups, if necessary, support with creation by agency (tip: agency can, for example, request information from partners and then finalize the layout, the group should provide the technical information), printing company
Additional cooperation partners (contract based):	If agency supports the project a contract will be required<br>• Printing company must be commissioned
Legal framework:	commissioning
Financing:	• Printing costs per card depending on the edition, rather manageable costs (max. 100 euros per card)<br>• Agency costs depending on the volume of support
Structure of cooperation among partners:	• Working group meeting<br>• Use plenary meetings for this<br>• Exchange via online communication platform<br>• By mail or conference calls
Frequency:	• i.e. annually
External representation	• Group decision, which topics should be when targeted<br>• In presentations<br>• Presentation at lectures and visits by interested parties<br>• Passing on to other networks and interested parties possible<br>• Presentation at congresses
Module Name:	**Outpatient Video-Supported Therapy**
Aim:	Outpatient adaptation of antiparkinson therapy
Strategic priorities:	• Real-life observation of the symptoms<br>• patient can create videos at any time<br>• call up exercise programs<br>direct telephone contact with patient
Participants among network partners:	• Resident neurologists and a neurological clinic / company: MVB Koblenz/ different health insurance companies (currently only BEK)
Additional cooperation partners (contract based):	• contract between all partners mentioned above required
Legal framework:	• Cooperation agreement (Kooperationsvertrag)
Financing:	• Health insurance
Structure of cooperation among partners:	1. Practice doctor evaluates videos alone<br>2. Practice doctor asks for support from the clinic<br>3. Clinic evaluates videos alone
Frequency:	once per year possible
External representation	currently very little presence, could be more
Module Name:	**Parkinson Nurse**
Aim:	Competent care and advice for Parkinson's patients (in Germany currently present in the stationary setting of the PANAMA network)
Strategic priorities:	• Qualification of nurses<br>• Increase employee commitment<br>Qualified care
Participants among networkpartners:	• Parkinson Nurses<br>• University Hospital
Setting	• University clinic with focus center<br>• (PANAMA Parkinson's ward)

Additional cooperation partners (contract based):	none
Legal framework:	none
Financing:	• participation fee for educational courses
Structure of cooperation among partners:	Nursing staff with additional qualifications are assigned to the appropriate ward area to care for Parkinson's patients.<br>• Parkinson's nurses help shape the Parkinson's Info Café
Frequency:	Permanent
External representation	/
Module Name:	**Standardized Treatment Path**
Aim:	• Development and implementation of a standardized intersectoral treatment path for Parkinson's patients with a focus on patients in need of specialized care<br>• Efficient, timely and equal care supply for all Parkinson patients, regardless of where they live and other sociodemographic factors
Strategic priorities:	• Consensus-based development of an intersectoral treatment path with the involvement of the regional outpatient sector<br>• Continuous path-based treatment with defined treatment content and a defined division of tasks and roles between specialized centers and the regional outpatient sectors<br>• Consideration of national and international recommendations on treatment standards for Parkinson's patients (if available)<br>• Interval-triggered monitoring and minimum treatment requirements<br>• Patient-specific definition of the division of tasks between specialized centers and regional outpatient sector and the possibility of falling below the generally applicable minimum treatment standard<br>• Three-month automated patient-based monitoring for all patients, regardless of the individual patient specifications within the framework of the path-based treatment concept<br>• Personnel implementation: Intersectoral case manager ("Parkinson pilot") as a personal contact and organizer of path-based treatment<br>• Technical implementation: Intersectoral electronic path-supporting patient management and documentation platform<br>• Evaluation of structure, process and result quality<br>• Implementation of a controlled growth process by involving other professional groups and partners
Participants among network partners:	• **Clinics:**<br>o Elbland Clinic Meißen<br>o Clinic at the Tharandter Wald Hetzdorf<br>o University Hospital Carl Gustav Carus Dresden<br>• **Scientific institutes , companies**<br>o Institute for Applied Computer Science eV, Leipzig (InfAI)<br>o Institute for Information Systems, TU Dresden<br>o What do I have? gGmbH<br>o Professorship for Movement Sciences, Chemnitz University of Technology<br>o Center for Medical Informatics, TU Dresden (ZMI)<br>• **Evaluation**<br>o Center for Evidence-Based Health Care (ZEGV), TU Dresden in cooperation with<br>o AOK PLUS Saxony-Thuringia<br>o Ikk Classic<br>• **Associations, professional organizations**<br>o German Parkinson's Association (dPV)<br>o Association of Statutory Health Insurance Physicians in Saxony (KVS)<br>• Saxon State Medical Association (Slaek)
Additional cooperation partners (contract based):	see above, with the exception of dPV , Slaek , AOK PLUS and ikk Classic (all associated cooperation partners without their own budget)
Legal framework:	Health services research project
Financing:	German Fund for coal exit<br>Combined funding from the Federal Ministry of Health and the Free State of Saxony
Structure of cooperation among partners:	As a module of PANOS: partnership research project, written cooperation agreement

Frequency:	Need-based
External representation	Web presence under construction
Module Name:	**Structured Patient School according to the self-management concept**
Aim:	• Immediately: Uniform training of Parkinson's patients to strengthen participation and autonomy as part of the health care research project Parkinsonnetzwerk Ostsachsen (PANOS)<br>Perspective: Together with national partners Establishment of a Germany-wide certifiable standardized structured patient school for Parkinson's patients
Strategic priorities:	Implementation strategy as a module of the Parkinson's Network East Saxony (PANOS) :<br>• Adaptation of the concept of the Swedish National Parkinson School for regional use in East Saxony<br>• Implementation of standardized patient training as usual for other chronic complex diseases (e.g. diabetes mellitus, asthma)<br>• Modular classroom training concept with 8-9 modules for small groups of patients and relatives<br>• Combination of knowledge transfer AND psychoeducative measures to implement a self-induced behavior modification<br>• Evaluation of immediate and delayed training concepts<br><br>Perspective e: Germany-wide standardized patient school for Parkinson's patients:<br>• Together with national cooperation partners, establishment of a nationwide standardized patient school for Parkinson's patients<br>• Modular implementation strategy<br>1. Needs assessment (patients: supra-regional focus groups, experts: multi-level consensus process, eg Delphi)<br>2. Development of a modern modular training concept, including consideration of innovative digitization components, formative evaluation<br>3. Summative evaluation<br>4. Development of certification process, continuous quality management<br>5. Comprehensive implementation by certification holders<br>• Module 1 started, planned for module 2 and subsequent joint research proposal
Participants among network partners:	Adaptation in the context of PANOS:<br>• Swedish National Parkinson School<br>• Department of Neurology, University Hospital Carl Gustav Carus Dresden<br>• Department of Neurology, Elbland Clinic Meißen<br>• Clinic at the Tharandter Wald Hetzdorf<br>• What do I have? gGmbH (electronic patient letter)<br>• Institute for Applied Health Services Research GmbH (inav), Berlin<br>• Evaluation: Center for Evidence-Based Health Care (ZEGV) TU Dresden<br><br>Germany-wide standardized patient school:<br>• Needs analysis (focus groups, expert-based Delphi method):<br>• Medical Psychology Neuropsychology and Gender Studies & Center for Neuropsychological Diagnostics and Intervention (CeNDI), University Hospital Cologne<br>• Department of Neurology, University Hospital Carl Gustav Carus Dresden<br>• Department of Neurology, Marburg University Hospital<br>• Neurological Clinic, Ruhr University Bochum<br>• Department of Neurology, Münster University Hospital<br>• Other participants and partners are welcome!
Additional cooperation partners (contract based):	• As a module of PANOS: partnership research project, written cooperation agreement<br>• Nation-wide patient school: No contractual commitment so far
Legal framework:	• PANOS: Health services research project<br>• Germany-wide patient school, needs assessment: Collaborative research project
Financing:	German Fund for coal exit<br>= Combined funding from the Federal Ministry of Health and the Free State of Saxony<br>• Module 1: Needs assessment: Industry funding, project application submitted to the German Parkinson's Association (dPV)<br>• Module 2 and more: Community application for third-party funding planned
Structure of cooperation among partners:	• PANOS: Partnership health services research project, written cooperation agreement<br>• nation-wide patient school: national research cooperation

Frequency:	• Need-based
External representation	• Through joint applications to dPV and via industrial partners
Module Name:	**PASS (Parkinson Assistant) Training**
Aim:	Qualified, Parkinson-experienced medical assistan7
Strategic priorities:	Curricular training consisting of basic and advanced courses nationwide
Participants among networkpartners:	medical assistant, sponsoring by pharmaceuticals or self-payers (practices)
Additional cooperation partners (contract based):	QUANUP e.V., Pharma, Practice owner
Legal framework:	Training contract / sponsorship contract
Financing:	Company sponsors the training for a certain number of participants. Practices pay per participating medical assistant
Structure of cooperation among partners:	QUANUP e.V. provides speakers. Basic course on weekends (Fri/Sat on 2 weekends; 1 advanced course (on Sat.) approx. 6 months later
Frequency:	Approx. 2-4 courses / year in different regions
External representation	Website quanup.de
Module Name:	**Parkinson Academy**
Aim:	Practical training of specialists (doctors, nurses, therapists)
Strategic priorities:	• Expansion of specialist knowledge with cooperation partners<br>• Increase attractiveness as referrer
Participants among networkpartners:	• Doctors university hospital<br>• Parkinson's Nurses University Hospital<br>• Settled neurologist<br>• Clinic neurologist<br>• Members of other specialist groups (assistants, therapists, depending on the focus)
Setting	• Max 15 people<br>• Practical training<br>• Very short theoretical update on current news<br>• Keynote speech on the main topic<br>• Practical exercise on a key topic where possible<br>• Bedside teaching
Additional cooperation partners (contract based):	none
Legal framework:	none
Financing:	Sponsorship
Structure of cooperation among partners:	Invitation via flyer, homepage<br>Registration required
Frequency:	once per year
External representation	/
Module Name:	**Deep Brain Stimulation (DBS) Consultation**
Aim:	Advising Parkinson's patients on deep brain simulation
Strategic priorities:	Individual advice and risk assessment regarding the indication deep brain simulation
Participants among network partners:	• Practice owner<br>• University hospital

	(i.e. Network Düsseldorf: Dr. med. Lorrain & Prof. Dr. med. Vesper (Head of Stereotaxy and Deep Brain Simulation, University Clinic Düsseldorf)
Setting	• /
Additional cooperation partners (contract based):	• no contract
Legal framework:	none
Financing:	none
Structure of cooperation among partners:	Joint consultation with practice owner + neurosurgeon with patient and support<br>Detailed individual clarification taking into account the individual course<br>communication: Telephone agreement between the practice and the secretariat
Frequency:	/
External representation	Internal communication in practice and the self-help groups
Module Name:	**Parkinson Board**
Aim:	Case discussion of complex patients
Strategic priorities:	• Professional Exchange<br>Strengthen team communication, with referrers
Participants among network partners:	• Parkinson expert's university clinic (doctors, nurses, therapists)<br>• Resident neurologist
Setting	University clinic with focus center<br>• Registration of a patient by a resident neurologist<br>• Discussion of casuistic on the Parkinson Board<br>• • Submission of specific diagnostic and / or therapeutic recommendations
Additional cooperation partners (contract based):	no contract
Legal framework:	none
Financing:	none
Structure of cooperation among partners:	After registration by email / phone, the case / question will be discussed in the presence of the neurologist or in absentia on the interdisciplinary board.<br>A written recommendation on the further procedure is then given to the neurologist.
Frequency:	By appointment, possibility of presentation in a weekly team meeting (i.e. Fridays 10.30 a.m.)
External representation	/
Module Name:	**Parkinson´s Info-Café**
Aim:	Informal exchange patients, relatives & Parkinson Nurses
Strategic priorities:	• informal exchange<br>• Advice on nursing aspects of the disease<br>Patient loyalty to the focus center
Participants among network partners:	• Parkinson Nurses University Hospital<br>• Patients<br>• Relatives
Setting:	• Forest restaurant with café<br>• Coffee & cake free of charge<br>• Informal exchange<br>• Occasionally a fixed program item (eg reading, presentation of new study, presentation of new device)
Additional cooperation partners (contract based):	none

Legal framework:	none
Financing:	none, donations from participants
Structure of cooperation among partners:	Invitation via flyer, local press<br>Registration required
Frequency:	Twice a year
Module Name:	**Virtual Visit ´s**
Aim:	Electronic consultation of complex patients
Strategic priorities:	• Assignment binding<br>• Simplified communication
Participants among network partners:	• Parkinson expert (university clinic)<br>• Resident neurologist<br>• Patient
Setting:	• University clinic with special focus on Parkinson's with video camera<br>• Practice rooms of the resident neurologist with video camera<br>• Both partners are registered with a certified video consultation provider<br>• The patient's consent is obtained before the start
Additional cooperation partners (contract based):	none
Legal framework:	Requirements for video consultation according to the E-Health Act
Financing:	no
Structure of cooperation among partners:	After prior registration by phone, an appointment will be made for an electronic consultation. In this, the specialist from the university clinic is introduced to the patient by the resident neurologist and the (complex) question of diagnostics and / or therapy
Frequency:	By arrangement
Module Name:	**Multimodal Complex Treatment**
Aim:	Therapy of motor and non-motor symptoms of Parkinson's disease
Strategic priorities:	• Increase patient safety and satisfaction<br>• Patient loyalty<br>• Representation as a center of competence
Participants among network partners:	• patient<br>• A GCT University Hospital<br>• Nursing staff (university clinic)<br>• Therapist University Clinic:<br>o physical therapy<br>o Occupational therapy<br>o Music therapy<br>• Speech therapy
Setting:	• At least 16 days inpatient treatment Multimodal assessment at the beginning / end of treatment<br>• 7.5 hours of therapy per week<br>• Interdisciplinary team meeting once a week
Additional cooperation partners (contract based):	no
Legal framework:	none
Financing:	Reimbursement via German health insurance system;<br>Billing via DRG , OPS code 8-97d
Structure of cooperation among partners:	Termination via case management, advanced notice required
Frequency:	Not applicable

Module Name:	**Rehab**
Aim:	Competence expansion of DBS external rehabilitation clinics
Strategic priorities:	• Increase the skills of external partners in dealing with DBS<br>Increase patient safety and satisfaction
Participants among network partners:	• Neurologist in rehabilitation clinic<br>• GCT University Hospital
Setting:	• Visit to an external rehabilitation clinic<br>• Training of doctors<br>• Advice on individual patients<br>• Telephone exchange for problematic cases
Additional cooperation partners (contract based):	no
Legal framework:	none
Financing:	no
Structure of cooperation among partners:	Personal appointment<br>Patients are invited by external partners
Frequency:	Depending on the institution site training approximately 1 x per year<br>Personal consultation as required
Module Name:	**Parkinson Summer Festival**
Aim:	Informal exchange of patients, relatives and specialists Information about current therapies<br>Information about current studies<br>Recruitment of study patients
Strategic priorities:	• Patient loyalty<br>• Representation as a competence center & research-active institution
Participants among network partners:	• Patient<br>• Relatives<br>• Doctors university hospital<br>• Students/ PhD students<br>• Parkinson's Nurses University Hospital Therapist university clinic
Setting:	• 150-200 participants<br>• Summer party with a mix of lectures, courses, research village, Parkinson's cinema<br>• Duration: 8 hours
Additional cooperation partners (contract based):	none
Legal framework:	none
Financing:	Sponsorship, donations
Structure of cooperation among partners:	Invitation via flyer, website
Module Name:	**Parkinson Symposium**
Aim:	Scientific training of doctors
Strategic priorities:	• Expansion of specialist knowledge among doctors<br>• Representation as a research-active institution /opinion-forming center
Participants among network partners:	• Doctors university hospital<br>• External doctors
Setting:	• 150-200 participants<br>• Lecture event with focus topic<br>• Duration: 8 hours

Additional cooperation partners (contract based):	none
Legal framework:	none
Financing:	Sponsorship
Structure of cooperation among partners:	Invitation via flyer, website<br>Registration required
Frequency:	every two years
